# Computer-assisted open reduction internal fixation of intraarticular radius fractures navigated with patient-specific instrumentation, a prospective case series

**DOI:** 10.1007/s00402-021-03856-6

**Published:** 2021-03-14

**Authors:** F. A. Casari, S. Roner, P. Fürnstahl, L. Nagy, A. Schweizer

**Affiliations:** 1 Orthopedic Department, Balgrist University Hospital, University of Zurich, Forchstrasse 340, 8008 Zürich-CH, Switzerland; 2 ROCS; Research in Orthopedic Computer Science, Balgrist University Hospital, University of Zurich, Balgrist-Campus, Lengghalde 5, 8008 Zurich-CH, Switzerland

**Keywords:** Patient-specific instrument, PSI, Computer-assisted surgery, Intra-articular radius fracture, 3D printing

## Abstract

**Background:**

Intra-articular fractures are associated with posttraumatic arthritis if inappropriately treated. Exact reduction of the joint congruency is the main factor to avoid the development of arthrosis. Aim of this study was to evaluate feasibility of computer-assisted surgical planning and 3D-printed patient-specific instrumentation (PSI) for treatment of distal intraarticular radius fractures.

**Method:**

7 Patients who suffered a distal intraarticular radius fracture were enrolled in this prospective case series. Preoperative CT-scan was recorded, whereupon a 3D model was computed for surgical planning and design of PSI for surgical navigation. Postoperative accuracy and joint congruency were assessed. Patients were followed-up 3, 6 and 12 months postoperatively.

**Results:**

Mean follow-up was 16 months. Over all range of motion was restored and flexion, extension and pronation showed significant recovery, *p* < 0.05. Biggest intraarticular joint step-off and gap reduced from average 2.49 (± 1.04) to 0.8 mm (± 0.44), *p* < 0.05 and 6.12 mm (± 1.04) to 2.21 mm (± 1.16), *p* < 0.05. Average grip strength restored (3–16 months) from 20.33 kg (± 7.12) to 39.3 kg (± 19.55) *p* < 0.05, 100% of the healthy contralateral side. 3D-accuracy for guided fragments was 2.07 mm (± 0.64) and 8.59° (± 2.9) and 2.33 mm (± 0.69) and 12.86° (± 7.13), *p* > 0.05 for fragments reduced with ligamentotaxis.

**Conclusion:**

Computer-assisted and PSI navigated intraarticular radius fracture treatment is feasible, safe and accurate. The benefits of this method, however, do not outstand the additional effort.

**Level of evidence:**

IV.

**Supplementary Information:**

The online version contains supplementary material available at 10.1007/s00402-021-03856-6.

## Introduction

Distal radius fractures are among the most common overall injuries accounting for one-fifth of all fractures in the emergency department [1]. Over lifetime the incidence increases with age and has a significant impact on general health and well-being [2, 3]. Women have a 15% lifetime risk to suffer a radius fracture while this risk decreases to 2% in men [4]. The literature provides evidence of the importance of restoring the radiocarpal joint surface as accurate as possible after trauma. Disharmonized gliding of the articular surface due to posttraumatic steps or gaps in the joint surface will cause osteoarthritis over the years [5–7]. In a biomechanical cadaver study, Baratz et al. discovered a fourfold increase of overloaded joint area at 1 mm step-off and even eightfold increase after 2 mm with peak pressure in the fracture line [8]. Therefore, exact reduction to reconstruct the joint surface cannot be overemphasized to prevent the development of osteoarthritis [9].

Computer-assisted approaches have proven as an excellent tool for difficult geometrical assessment, surgical planning and navigation of the procedure using 3D-printed patient-specific instruments (PSI). For elective corrective procedures, such tools are well established in hand surgery [10–15]. Recent studies evaluating computer assisted distal intraarticular radius corrections have shown favorable results for secondary reconstruction of joint surfaces healed in malposition [13, 16]. At this point, grinding of the incongruent joint over time might have caused further damage to the cartilage.

This prospective study was conducted treating acute intraarticular radius fractures with computer-assisted planning and executed using 3D-printed patient-specific instrumentation. Purpose of this study was to evaluate feasibility, anatomic reconstruction and short-term functional outcome of acute intraarticular radius fractures treated with this approach.

## Methods

In this prospective study conducted from September 2017 to March 2020, seven patients with distal intraarticular fractures of the radius requiring surgical treatment were enrolled and underwent computer-assisted open reduction internal fixation navigated with 3D-printed PSI. Minimal follow-up was 12 months. Study participants were at least 18 years old, had given informed consent and a clinical indication for computer tomography (CT). Exclusion criteria were additional shaft fracture, pregnancy, breastfeeding, contraindications such as tumors, use of addictive substances and allergies on polyamide. The study was reviewed and approved by the ethics committee of the canton of Zurich (Basec-Nr. 2016-01925).

### Evaluation

Patients were followed-up clinically 3, 6 and 12 months postoperatively and range of motion (ROM) (flexion, extension, ulnar deviation, radial deviation, supination and pronation) and grip strength (JAMAR in kg; Sammons Preston, Bolingbrook IL, USA) were assessed. Three months after surgery a CT scan was recorded for evaluation of bone consolidation, procedure accuracy and joint congruency. The non-normally distributed data were statistically tested using the Wilcoxon-Rank test (accuracy, joint congruency) and ANOVA test (ROM, grip strength).

### Sequence

Patients were enrolled during their emergency visit and CT scan (slice thickness, 1 mm; 120 kV; Philipps Brilliance CT) of the injured hand for fracture evaluation and of the healthy contralateral hand as template for procedure planning was recorded.

**Fig. 1:**
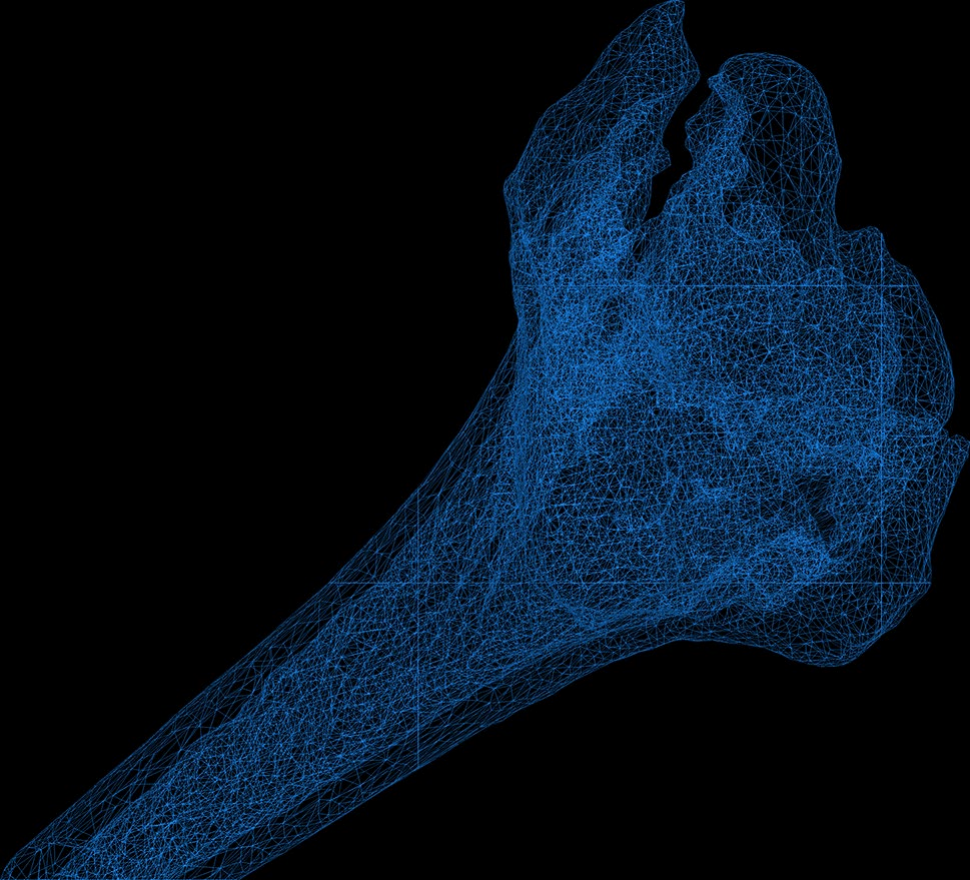
3D model of a intraarticular fractured radius, reconstructed in a triangular mesh

The scans were reconstructed to a three-dimensional (3D) triangular surface mesh (Fig. 1) using region growing and the marching cubes algorithm [17].

**Fig. 2:**
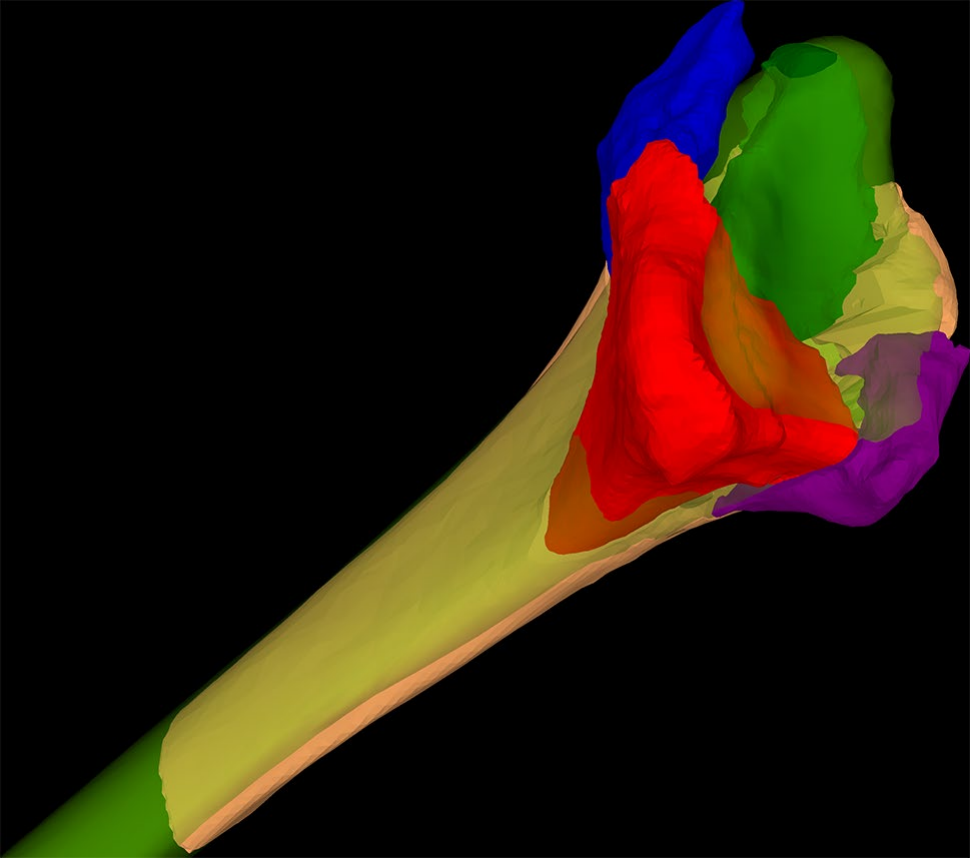
3D model of fractured radius, the intraarticular fragments are labeled in blue, red, green and purple. The template model is labeled in semitransparent green

Those models were then uploaded into our in-house developed planning software CASPA (Computer-Assisted-Surgery-Planning-Application). The healthy contralateral hand served as planning template and was mirrored and superimposed to the model of the fractured radius. To allow a precise superimposition of the 3D-models (fractured and healthy template), the surface is registered via the iterative closest point (ICP) method (Fig. 2). This allows to assess the fracture visually in 3D and analyze fragment displacement [18–20].

**Fig. 3:**
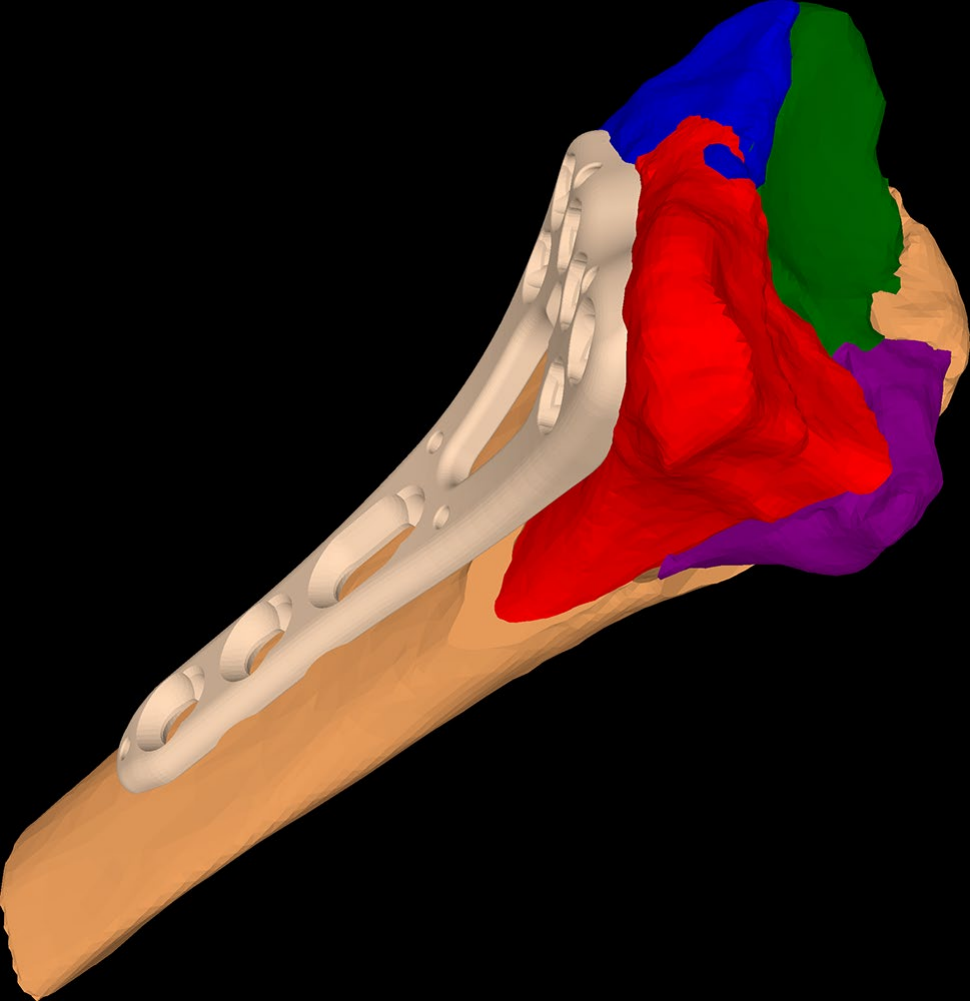
3D model of anatomically reduced fragments and osteosynthesis plate

**Fig. 4:**
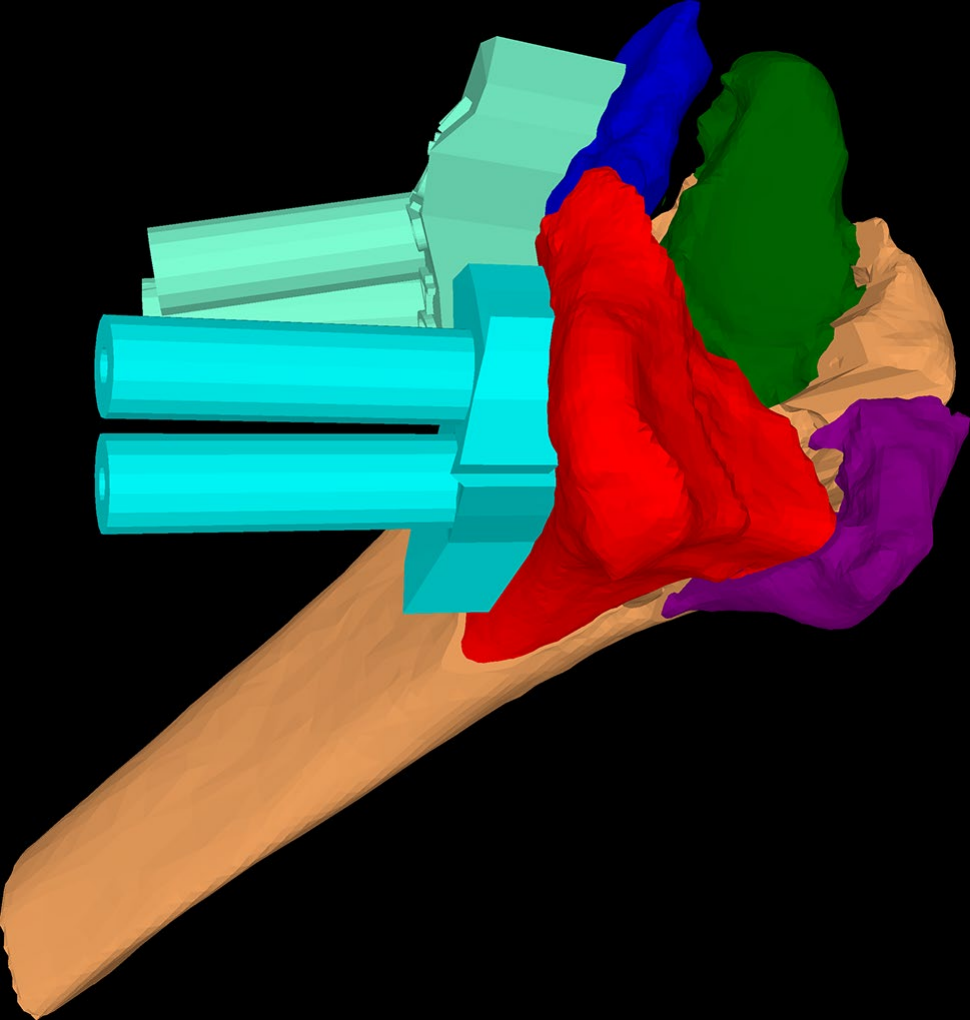
3D model of fractured radius with two PSI labeled in cyan blue designed for anatomical reduction

With the surgical planning software, the procedure, reduction and plate positioning were manually planned by trial and error by placing the fragments and osteosynthesis plate in to the reduced position (Fig. 3). When a satisfactory anatomical reduction plan was achieved, PSI for navigation of fragment reduction was designed (Fig. 4). This step required interdisciplinary cooperation of the performing surgeon and a biomechanical engineer.

**Fig. 5:**
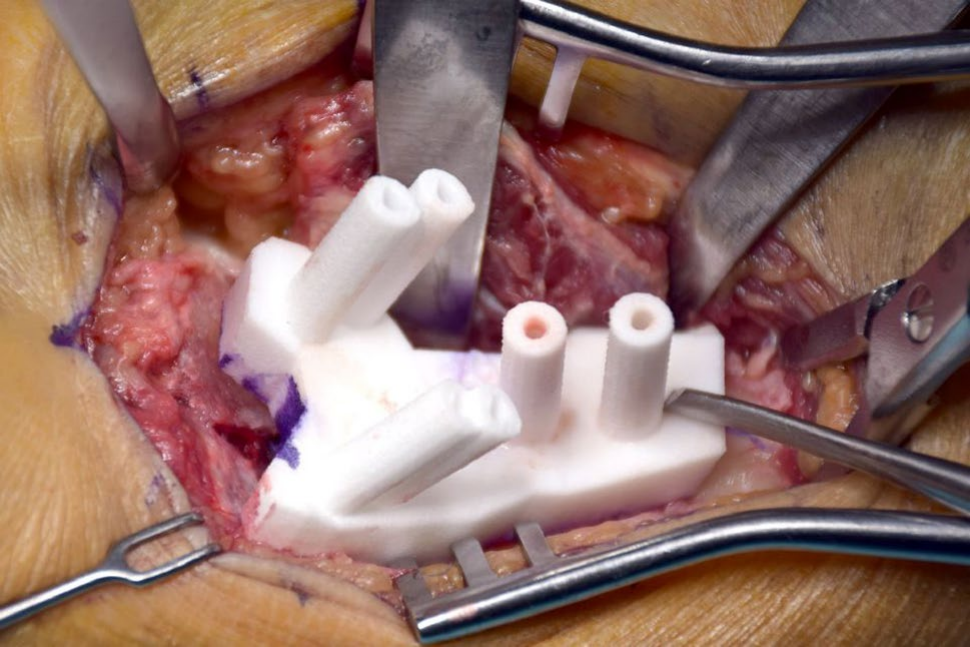
Intraoperative footage of an intraarticular fracture with PSI. The guide drillholes allow reduction and securing of the fragments with k-wires

The surgical procedures were executed by two senior hand surgeons, A.S and L.N. All fractures were surgically accessed and plated from a palmar exposure, as the palmar access allows PSI positioning with less interference of the surrounding soft tissue, i.e. neurovascular structures as tendons (Fig. 5).

Therefore, the dorsal fragments could not be PSI-guided and were repositioned secondarily via ligamentotaxis.

For accuracy evaluation, the postoperative CT-scan was imported into CASPA as described. Then, the outcome model was superimposed to the preoperative plan. The residual plan to outcome error was measured for each fragment in reference to the 3D-coordinate system assessing transition and rotation in all three axis (*x*, *y*, *z*). For comprehensibility these 3 + 3 values in regard to each axis of the coordinate system were converted into a 3D angle and 3D transition in regard to the 3D-coordinate system. To determine articular congruency, the biggest preoperative step-off and gap was determined visually in the 3D model and planar CT imaging. The same defect was then measured in the conventional CT slices (pre- and postoperative scan) from the fragment edges and grading according to Knirk and Jupiter’s articular congruency grading [7].

## Results

A total of seven patients were included in this study. Mean age was 47.71 years, four injured their dominant hand, mean procedure time was 142 min with 02:58 min fluoroscopic time. The demographic data and AO fracture type are given in Table 1.

**Table 1:** Listing of the cases with sex, age, pathologic and dominant side, time to ER-visit, occupation, back to work and AO classification

Case	Sex	Age, years	Pathologic side	Dominant side	Time ER-visit to surgery, days	Occupation	Back to work	AO classifcation
1	M	18	Right	Right	2	Apprentice (office)	Yes	C3
2	M	74	Left	Right	2	Retired chaffeur	n.a	C3
3	F	64	Left	Left	3	Retired secretary	n.a	C3
4	F	54	Right	Right	3	Housekeeper	Yes	C3
5	M	36	Left	Right	3	Maintainance technician	Yes	C1
6	M	40	Left	Right	7	Computer scientist	Yes	C2
7	M	48	Right	Right	5	Strategy consultant	Yes	C2

n.a.  not applicable

All patients had early bony consolidation after 3 months except one patient who was a heavy smoker (case 5). He developed pseudarthrosis, which was revised with iliac crest autograft and showed secondary healing in succession.

Evaluation of range of motion and joint congruency is given in Table 2. Biggest intraarticular joint step-off and gap reduced from average 2.49 (± 1.04) to 0.8 mm (± 0.44), *p* < 0.05 and 6.12 mm (± 1.04) to 2.21 mm (± 1.16), *p* < 0.05. In all except one patient (case 5), joint congruency was reconstructed to grade 0 when graded by Knirk and Jupiter’s articular congruency grading [7].

**Table 2:** Evaluation of range of motion, grip strength and joint congruency

Case	Flexion, (°)	Extension, (°)	Pronation, (°)	Supination, (°)	Grip strenght/kontralateral (JAMAR, kg)	Case	Step-off, (mm)	Gap, (mm)
1 (3 mo)	20	30	80	90	16/30	1 (before)	3.04	7.2
1 (12 mo)	60	55	90	90	28/30	1 (after)	0.66	1.1
2 (3 mo)	60	50	70	70	27//43	2 (before)	1.35	12
2 (12 mo)	Lost to f.u	Lost to f.u	Lost to f.u	Lost to f.u	Lost to f.u	2 (after)	0.5	4.4
3 (3 mo)	25	40	70	55	22/24	3 (before)	1.68	4.8
3 (12 mo)	45	70	80	75	28/24	3 (after)	0.8	2.3
4 (3 mo)	25	10	25	20	13/21	4 (before)	3.23	3.6
4 (17 mo)	40	25	50	45	21/21	4 (after)	0.94	2.3
5 (3 mo)	30	60	80	40	30/50	5 (before)	4.06	3.3
5 (25 mo)	40	70	80	40	46/50	5 (after)	1.71	2.5
6 (6 mo)	30	40	60	40	30/50	6 (before)	1.39	1.8
6 (12 mo)	55	60	65	75	42/47	6 (after)	0.43	0.9
7 (3 mo)	50	40	70	35	14/44	7 (before)	2.7	9.6
7 (12 mo)	65	70	75	90	75/44	7 (after)	0.58	2

f. u. follow-up

**Fig. 6:**
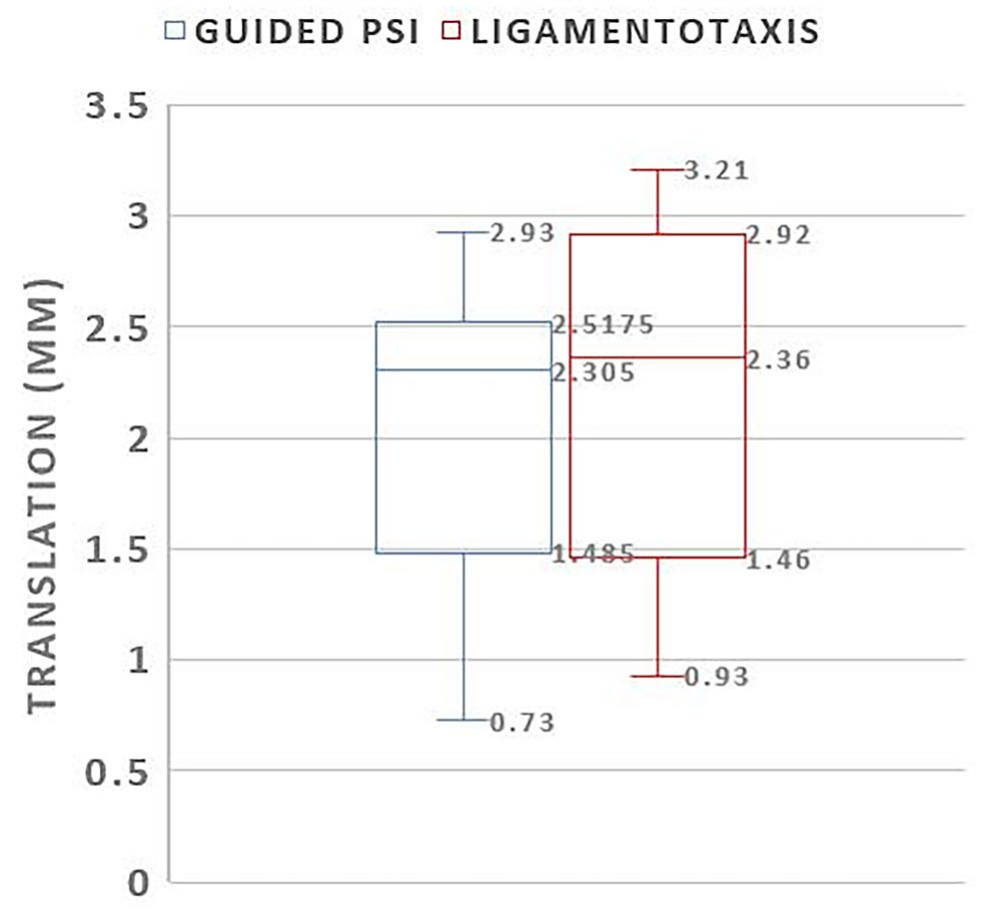
Box-plot comparing translational error in millimeters of PSI guided and ligamentotaxis reduced fragments

**Fig. 7:**
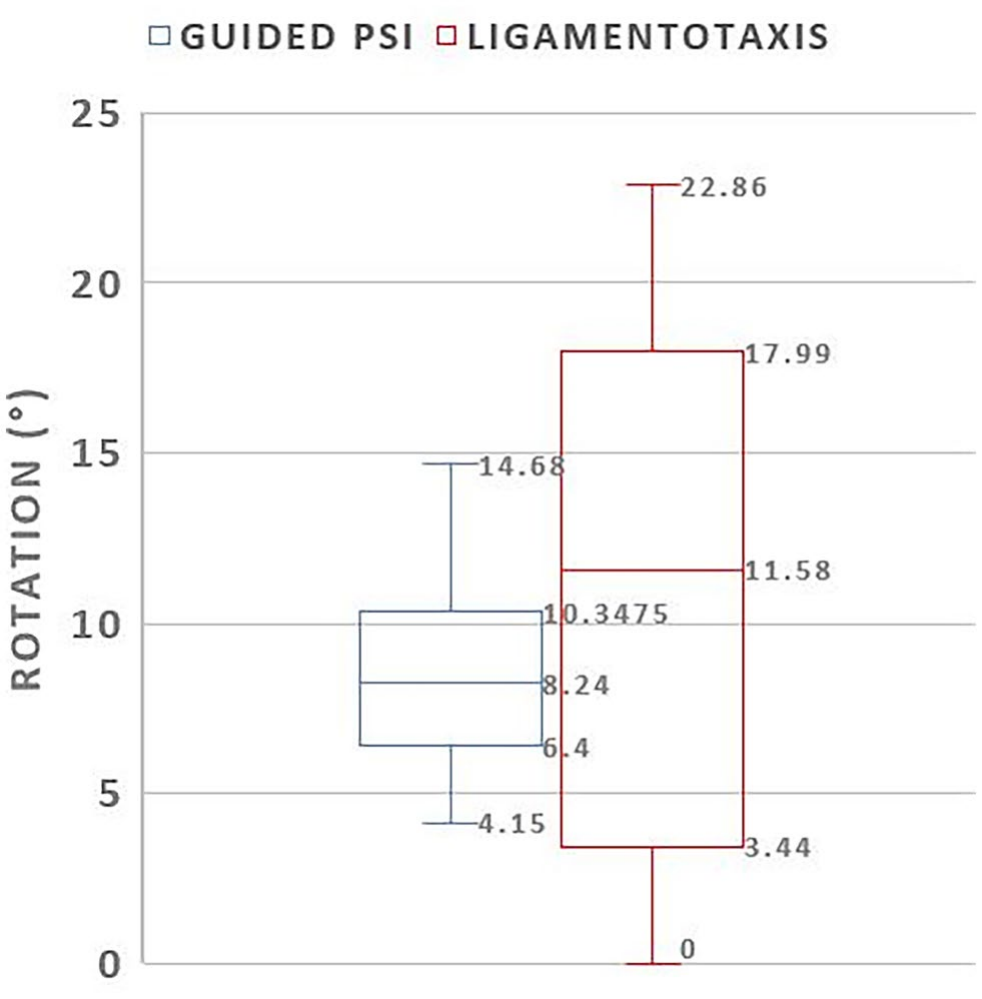
Box-plot comparing rotational error in degrees of PSI guided and ligamentotaxis reduced fragments

Average grip strength recovered (3–16 months) from 20.33 (± 7.12) to 39.3 kg (± 19.55) *p* < 0.05, 100% of the healthy contralateral side. The osteosynthesis plate had an average plan to outcome error of 1.6 mm (± 0.86) and 0.86° (± 1.38). Residual surgical plan to outcome error for the guided fragments is compared to the fragments reduced with ligamentotaxis in Figs 6 and 7. Procedure-specific data are given in Table 3.

**Table 3:** Procedure specific data: anesthesia, time, arthroscopy, plate type, follow-up, plan to outcome error, fragments, plate removal, bone graft

Case	Anesthesia	Procedure time (min)	Additional arthroscopy	Plate type	Follow-up, months	3D-angle, (°), LT	3D-angle, (°), PSI	3D-translation, (mm), LT	3D-translation, (mm), PSI	Number of fragments/PSI guided	Plate removal	Bone graft
1	Nerve block	168	Yes	Arthrex 8916VSR-05 (std)	12	8.73	11.37	2.64	2.42	4/2	Yes	No
2	Nerve block	107	No	Synthes-VA-LCP 2.4–2.7, 2 collumn	12	17.32	5.84	2.6	2.76	3/1	No	No
3	Nerve block	155	Yes	Synthes-VA-LCP 2.4–2.7, 2 collumn	12	12.92	7.98	2.49	2.4	4/3	No	No
4	Nerve block	209	Yes	Synthes-VA-LCP 2.4–2.7, 2 collumn	17	22.86	7.54	2.05	1.14	3/2	Yes	No
5	Nerve block	121	Yes	Synthes-VA-LCP 2.4–2.7, 2 collumn	25	–	7.56	–	2.21	1/1	No	Yes
6	Nerve block	102	No	Arthrex-8916VWR-03 (wide)	12	6.655	8.92	2.01	2.49	2/2	Yes	No
7	Nerve block	136	Yes	Synthes-VA-LCP 2.4–2.7,2 collumn	12	–	11.03	–	1.29	2/2	Yes	No

## Discussion

This was a feasibility study evaluating the use of computer-assisted open reduction internal fixation of distal intraarticular fractures, navigated with PSI. For acute fracture treatment, 3D printing has proven as a valuable support. Previous studies have evaluated 3D-printed models of the fractured bone for better geometric understanding intraoperatively. Simple real-size models proved as valuable for better understanding fracture patterns in different bones [21].

Chen et alcompared two groups, one with the use of 3D-printed models of the fractured radius and one with conventional surgical treatment [22]. The simple model for geometric understanding of the fracture led to reduced use of fluoroscopy, blood loss and operation time. the functional outcome, however, showed no significant difference in range of motion.

The presented method is a further development of the 3D-printing method for acute fracture treament. The study team designed specific 3D-printed PSI, which have proven as accurate navigation tools. As of yet PSI have only been used for elective corrective procedures when malunion occurred after trauma or to adress other bone deformities [10, 11, 13, 23, 24]. This study showed that the use of computer-assisted open reduction internal fixation using PSI is an accurate, safe and feasible method.

The focus of this study was to analyze the quality of fragment reduction and restoration of joint congruency. Concerning accuracy and anatomic reconstruction the results favor the method. It has to be noted, however, that the current literature indicates no clinical association in regard of symptoms, pain and hand function and radiographic presence of posttraumatic arthrosis [5, 6].

Knirk and Jupiter [7] presented a study of conventionally treated intraarticular fractures [7]. Although 65% of the included patients showed radiographic signs of arthritis, (nearly all patients (93%) were symptomatic considering pain. On the other hand, loss of bone length was described as a factor influencing grip strength. This malformation was not seen in any patients as analyzed in the postoperative 3D model. The most important factor regarding the development of posttraumatic arthritis is insufficient reconstruction of joint congruity [7]. Knirk and Juptier defined residual joint incongruency over 2 mm as a risk to develop arthrosis [7]. Therefore, the presented results with an average resdiual step-off of 0.8 mm might indicate good results regarding the development of arthritis. The residual joint gap was in average 2.21 mm; however, the CT evaluation could not provide information if the gap was filled with scar and/or fibrotic cartilage tissue resulting in intact articular surface.

Catalano et al. presented a study of a case series of young adults below the age of 45 years treated with conventional open reduction internal fixation [5]. The group found a strong correlation between residual joint incongruency, with an average maximum step-off of 1.6 ± 1.2 mm, and 1.5 ± 1.6 mm gap displacement and the development of posttraumatic osteoarthrosis in the course of 5.5 years. The radiographic presence of posttraumatic joint degeneration, however, did not lead to poor functional outcomes.

The same study group with Goldfarb et al. reported another follow-up of 15 years after surgery [6]. In 13 of the 16 patients arthrosis was present in the wrist joint with joint space narrowing. Again, the patients maintained a high level of function even with radiologic signs of postraumatic arthritis.

Ono et al. reported a postoperative step-off of 1 mm and more in 21% (15/70) and 2 mm or more in 7% (5/70) of patients treated with open reduction internal fixation [25]. The presented PSI method had one case 14% (1/7) with a postoperative step-off over 1 mm and no cases above 2 mm. The additional accuracy of joint reconstruction achieved using PSI seems to have no influence on the functional outcome according to the literature [5, 6, 25, 26]. Further long-term follow-up will answer the question concerning the development of further postraumatic degeneration.

Schweizer et al. presented a comparable study of six patients for elective PSI corrected malunions of intraarticular fractures. Recovery of range of motion was similar after 1 year [13]. Also, surgical accuracy of restoration of joint congruency and rotational error (step-off 0.5, 0.9, 0.3, 0.5, 1.4, 0.6, 1.0 mm and 2, 8, 10, 3, 10, 2, 8°) was very similar to the presented values in this patient cohort.

The statistical analysis of the primary guided fragments using PSI and the secondary guided fragments by ligamentotaxis showed no significant difference. This indicates that the navigated reduction provides also more accurate reduction for the ligamentotaxis reduced fragments.

The here presented method has obvious drawbacks. Patients have to undergo additional CT scanning of the healthy contralateral side, exposing them to extra radiation. The planning and PSI manufacturing adds extra costs approximately €150–€250 (USD $220–$320) [13] per case and time to the treatment process. Next, a significant limit of the PSI method is that only fragments that are sufficiently exposed can be guided with the PSI. The dorsal fragments could not be accessed via the palmar exposure. Previous classifications were only descriptive and did not indicate a specific surgical approach [27]. In a recent article, Hintringer et al. highlighted the relevance of considering the traumamechanism to determine the surgical approach, which can be in conflict with the standardized palmar acess for PSI use [28]. Regarding the rapid development of surgical navigation technology, an augmented reality-based application might advance the here presented approach allowing a traumamechanism-based surgical access [29, 30].

Another limitation of this study is the small sample size. Patients who did not consent to participate were concerned of additional time for planning and manufacturing time. This limited the interest of patients to participate in this study as most patients wanted early surgery. Also, we did not provide a conventional group for comparison. Patients would have had to undergo CT scanning solely for accuracy determination. This does not justify the additional radiation exposure.

## Conclusion

This study proved computer-assisted open reduction internal fixation navigated with patient-specific instruments as a feasible, safe and accurate treatment option. The benefits of this method, however, do not outstand the additional effort. The major improvement is exact evaluation and correction of rotational malposition of bone fragments. Symptomatic patients with intraarticular fractures healed in malpositioning therefore remain who benefit most of the computer assisted method.

## Supplementary Information

Below is the link to the electronic supplementary material.

Supplementary file1 (XLSX 5505 KB)
